# Transgenic *IDH2*^R172K^ and *IDH2*^R140Q^ zebrafish models recapitulated features of human acute myeloid leukemia

**DOI:** 10.1038/s41388-023-02611-y

**Published:** 2023-02-04

**Authors:** Dandan Wang, Lichuan Zheng, Bowie Yik Ling Cheng, Chun-Fung Sin, Runsheng Li, Sze Pui Tsui, Xinyu Yi, Alvin Chun Hang Ma, Bai Liang He, Anskar Yu Hung Leung, Xuan Sun

**Affiliations:** 1 Division of Haematology, Department of Medicine, LKS Faculty of Medicine, The University of Hong Kong, Pokfulam, Hong Kong SAR, China; 2 Department of Pathology, Queen Mary Hospital, Hong Kong SAR, China; 3 Department of Infectious Diseases and Public Health, City University of Hong Kong, Hong Kong SAR, China; 4 BGI-Genomics, BGI-Shenzhen, Shenzhen, China; 5 Department of Health Technology and Informatics, The Hong Kong Polytechnic University, Hung Hom, Hong Kong SAR, China; 6 Guangdong Provincial Key Laboratory of Biomedical Imaging and Guangdong Provincial Engineering Research Center of Molecular Imaging, The Fifth Affiliated Hospital, Sun Yat-sen University, Zhuhai, Guangdong 519000 China

**Keywords:** Acute myeloid leukaemia, Cancer genomics, Sequencing

## Abstract

Isocitrate dehydrogenase 2 (*IDH2*) mutations occur in more than 15% of cytogenetically normal acute myeloid leukemia (CN-AML) but comparative studies of their roles in leukemogenesis have been scarce. We generated zebrafish models of *IDH2*^R172K^ and *IDH2*^R140Q^ AML and reported their pathologic, functional and transcriptomic features and therapeutic responses to target therapies. Transgenic embryos co-expressing *FLT3*^ITD^ and *IDH2* mutations showed accentuation of myelopoiesis. As these embryos were raised to adulthood, full-blown leukemia ensued with multi-lineage dysplasia, increase in myeloblasts and marrow cellularity and splenomegaly. The leukemia cells were transplantable into primary and secondary recipients and resulted in more aggressive disease. Tg*(Runx1*:*FLT3*^ITD^*IDH2*^R172K^) but not Tg(*Runx1*:*FLT3*^ITD^*IDH2*^R140Q^) zebrafish showed an increase in T-cell development at embryonic and adult stages. Single-cell transcriptomic analysis revealed increased myeloid skewing, differentiation blockade and enrichment of leukemia-associated gene signatures in both zebrafish models. Tg(*Runx1*:*FLT3*^ITD^*IDH2*^R172K^) but not Tg(*Runx1*:*FLT3*^ITD^*IDH2*^R140Q^) zebrafish showed an increase in interferon signals at the adult stage. Leukemic phenotypes in both zebrafish could be ameliorated by quizartinib and enasidenib. In conclusion, the zebrafish models of *IDH2* mutated AML recapitulated the morphologic, clinical, functional and transcriptomic characteristics of human diseases, and provided the prototype for developing zebrafish leukemia models of other genotypes that would become a platform for high throughput drug screening.

## Introduction

Isocitrate dehydrogenases (IDH) are a group of enzymes that catalyze the conversion of isocitrate to α-ketoglutarate in the physiologic citrate acid cycle [1]. Mutations of *IDH* include *IDH1*^R132^, *IDH2*^R140^, and *IDH2*^R172^ in which arginine is substituted and they occur in more than 25% cases of acute myeloid leukemia (AML) with normal cytogenetics [2]. *IDH* mutations confer novel substrate specificity to the enzyme and instead of converting isocitrate to α-ketoglutarate, mutated IDH2 convert the latter to 2-hydroxyglutarate (2-HG). 2-HG is an oncometabolite and is associated with epigenetic alteration, genetic instability, and malignant transformation of hematopoietic cells [3–5]. Transgenic and knock-in mouse models of *IDH1*^R132^ and *IDH2*^R140Q^ mutated AML have been reported, showing that these mutations, singly or in combination with co-existing mutations, induced leukemogenesis [6–9]. However, animal models of mutated *IDH2*^R172K^ with clinicopathologic characteristics of human AML are scarce [6, 7, 10, 11].

Zebrafish has emerged as a model organism to study human diseases, including leukemia [12]. The optical transparency and high fecundity are distinct advantages, and the zebrafish genome and hematopoietic system are remarkably similar to those in mice and human [13]. Moreover, recent advances in genome editing, transgenesis, and rapid embryonic development have made zebrafish a unique model for studying mutation combinations at high throughput [14]. Over-expression of human *IDH1*^R132H^ has been shown to induce myelopoiesis in zebrafish embryos, suggesting that the pathogenetic pathway in *IDH* mutation is conserved in zebrafish [15, 16].

In this study, we established transgenic zebrafish models of *IDH2*^R172K^ that recapitulated clinicopathologic features of human AML. Comparative studies on *IDH2*^R140Q^ highlighted both similarities and differences between the two *IDH2* mutants in leukemogenesis. These models may provide important platforms for high throughput drug screening targeting *IDH* mutations in AML.

## Results

### Effects of human *IDH2* mutations on myelopoiesis

**Fig. 1:**
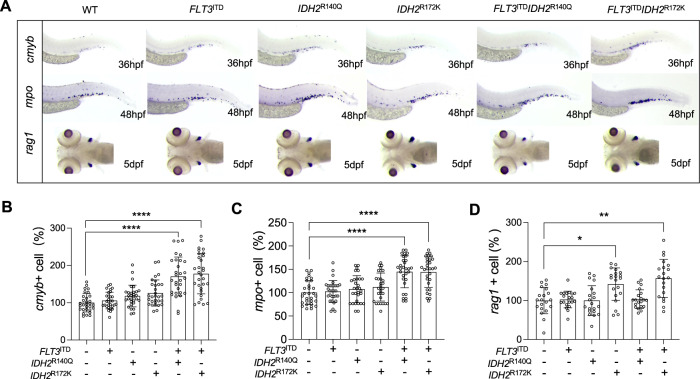
Lineage-specific expression of *IDH2* mutations exacerbated hematopoiesis and myelopoiesis in zebrafish embryos. **A** Brightfield images of WISH for *cmyb* at 36hpf, *mpo* at 48hpf, and *rag1* at 5dpf in the mutant (*n* = 20) and WT (*n* = 20) zebrafish embryos. **B** Quantification of *cmyb*, *mpo* (**C**), and *rag1* (**D**) respectively. The expression of each marker was presented as the percentage of each marker expressed in the mutant embryos relative to that of the WT embryos. Data are mean ± s.e.m and statistical analysis was performed by One-way Anova, *P < 0.05, **P < 0.01, ****P < 0.0001.

Zebrafish *idh2* exhibited remarkable similarities in amino acid sequence and syntenic neighboring genes to those of humans (Fig. S2A, B), suggesting orthologous relationships. To examine the effects of human *IDH2* mutations on myelopoiesis at the embryonic stage, *IDH2*^R172K^ and *IDH2*^R140Q^ mRNA were microinjected up to 200 pg into wildtype zebrafish embryos at 1-cell stage. Expression of *IDH2* mutations induced a marked increase in 2HG level, surrogate of mutant *IDH2* expression (Fig. S3A). Intriguingly, the equivalent amount of mRNA induced a substantially higher increase in 2HG in *IDH2*^R172K^ than in *IDH2*^R140Q^ injected embryos. Transient expression of *IDH2*^R172K^ or *IDH2*^R140Q^ mRNA had little effect on primitive myeloid progenitor as shown by whole mount in-situ hybridization (WISH) of *pu.1* (Fig. S3B, C); however, both *IDH2* mutations induced a remarkable increase in definitive hematopoietic stem and progenitor cells (HSPC) (*cmyb*) (Fig. S3B, D) and neutrophils (myeloperoxidase, *mpo* and Sudan Black B, SBB staining) (Fig. S3B, E, F). The prominent changes in embryonic myelopoiesis induced by *IDH2* mutations prompted us to generate transgenic zebrafish lines with stable expression of *IDH2* mutations in a lineage-specific manner. The generation of stable transgenic lines was described in “Materials and Methods” and Supplementary Information. F1 transgenic embryos were examined for hematopoietic gene expression by WISH and genotyped individually afterward. Interestingly, lineage-specific expression of *IDH2* mutations did not alter the abundance of HSPC or myeloid cells (Fig. 1A–C) in the embryos. On the other hand, only Tg(*Runx1*:*IDH2*^R172K^) but not Tg(*Runx1*:*IDH2*^R140Q^) embryos showed a significant increase in *rag1* (T-cell marker) expression compared with wildtype siblings (Fig. 1A, D). F2 double transgenic embryos were generated by crossing F1 Tg(*Runx1*:*IDH2*^R172K^) or Tg(*Runx1*:*IDH2*^R140Q^) to Tg(*Runx1*:*FLT3*^ITD^) [17] to investigate potential synergistic effects of these mutant genes which may co-exist in AML patients. Embryos co-expressing *FLT3*^ITD^ and *IDH2*^R140Q^ or *IDH2*^R172K^ showed a significant increase in markers associated with HSPC (*cmyb*, *runx1*) (Fig. 1A, B; Fig. S4A, B); neutrophils (*mpo*; SBB) (Fig. 1A, C; Fig. S4A, C) and pan-leukocyte marker *l-plastin* (Fig. S4A, D), when compared with siblings carrying single or no mutation. An increase in T-cell marker (*rag*1) (Fig. 1A, D) in the developing thymus was only seen in embryos carrying *IDH2*^R172K^ but not *IDH2*^R140Q^ irrespective of co-existing *FLT3*-ITD. Markers associated with primitive erythropoiesis (*gata1, hbae1.1*) and early myeloid progenitor (*pu.1*) were unaffected (Fig. S4A, E, F, G).

**Fig. 2:**
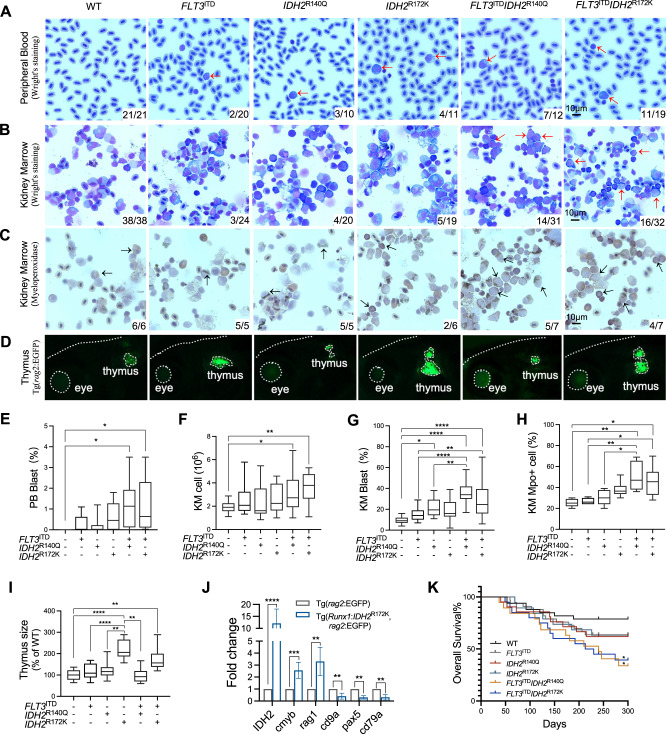
Development of AML-like disease in *IDH2* mutant zebrafish. **A** Representative Wright’s staining of the peripheral blood (PB) and KM cells (**B**), and myeloperoxidase staining of the KM cells (**C**) from the mutant zebrafish and WT siblings. The blast and Mpo+ cells are indicated by red and black arrows respectively. **D** Visualization of the thymus from the mutant and WT zebrafish via fluorescent microscopy, the edge of the fish head, thymus and eye was indicated by white dash lines in Tg(*rag2:*EGFP) background fish. **E** The percentage of the blast cell in the PB of the transgenic mutant zebrafish (*n* = 10) and WT siblings (*n* = 10). **F** The total cell number, the percentage of blast cell (**G**), the percentage of Mpo+ cells (**H**) in the KM of the transgenic mutant zebrafish (*n* = 6) compared with WT siblings (*n* = 6). **I** The relative size of the thymus in the transgenic mutant zebrafish (*n* = 9) compared with WT siblings (*n* = 9). **J** Relative mRNA expression of *IDH2, cmyb, rag1, cd9a, pax5, and cd79a* in the thymus of the *IDH2*^R172K^ (*n* = 3) and wildtype zebrafish (*n* = 3). **K** Overall survival of the WT (*n* = 33), single mutant (*n* = 33 for *FLT3*^ITD^, *n* = 21 for *IDH2*^R140Q^, *n* = 19 for *IDH2*^R172K^), and double mutant (*n* = 19 for *FLT3*^ITD^*IDH2*^R140Q^, *n* = 20 for *FLT3*^ITD^*IDH2*^R172K^) zebrafish. The numbers in the bottom right-hand corner in **A**–**C** indicated the number of fish with the characteristic phenotypes / the total number of fish in each group. Data are mean ± s.e.m. One-way Anova was performed for **E**, **F**, **G**, **H**, and **I**, *P < 0.05, **P < 0.01, ****P < 0.0001. Student’s *t* test was performed for **J**, **P < 0.01, ***P < 0.001, ****P < 0.0001. Log-Rank test was performed for **K**, *P < 0.05.

F2 larvae carrying single *IDH2* mutation or double mutations with *FLT3*^ITD^ were raised to adulthood. Morphologically, Tg(*Runx1*:*FLT3*^ITD^*IDH2*^R140Q^) and Tg(*Runx1*:*FLT3*^ITD^*IDH2*^R172K^) showed circulating blasts in peripheral blood (Fig. 2A, E). Kidney marrow (KM) showed dyserythropoietic features, characterized by the presence of bi-nucleated erythroid cells, nuclear-cytoplasmic asynchrony, and nuclear irregularity (Fig. 2B and Fig. S5). Neutrophils were dysplastic and morphologic features of pseudo-Pelger-Huet anomaly and hypogranularity, reminiscent of human myelodysplastic syndrome, were evident (Fig. 2B and Fig. S5). KM blasts showed abnormal morphology with highly irregular nuclear contours (Fig. 2B and Fig. S5).

Quantitatively, there were significant increases in total cell number per KM (Fig. 2F) and KM blast percentage [Tg(*Runx1*:*FLT3*^ITD^*IDH2*^R140Q^): Blasts = 35.6 ± 2.7%; Tg(*Runx1*:*FLT3*^ITD^*IDH2*^R172K^): Blasts = 28.9 ± 4.2%] (Fig. 2B, G), which were positive for Mpo, consistent with their myeloid lineage (Fig. 2C, H). To further quantify KM changes in these transgenic fish, they were crossed to Tg (*mpo*: EGFP), and EGFP signals were used to define the KM size (Fig. S6A). Both double transgenic mutant fish showed a significant increase in KM size (Fig. S6A, C). Furthermore, there was a significant increase in the size and cellularity of the spleen (Fig. S6B, D–F,), which was infiltrated by blasts and myelomonocytic cells (Fig. S6G).

The increase in *rag1* expression in F2 embryos carrying Tg(*Runx1*:*IDH2*^R172K^) and Tg(*Runx1*:*FLT3*^ITD^*IDH2*^R172K^) led us to examine T-cell development in adult fish. These embryos were raised to adulthood and crossed with Tg(*rag2*: EGFP) fish in which T-cells in the thymus expressed EGFP (Fig. 2D). The thymus of the adult transgenic Tg(*rag2:*EGFP*/Runx1:IDH2*^R172K^) and Tg(*rag2:*EGFP*/Runx1:FLT3*^ITD^*IDH2*^R172K^) fish were larger than the *IDH2*^R140Q^ counterparts (Fig. 2D, I). The enlarged thymus expressed mutated human *IDH2*^R172K^ (Fig. S6H) and showed an increase in *rag1* and *cmyb* (T-cell progenitors and common lymphoid progenitors) but a decrease in *cd79a*, *pax5,* and *cd9a* (B-cells) expression (Fig. 2J).

Double transgenic adult fish also showed a shortened survival after 100 days of age [Median survival: WT: not reach; single mutant: not reach; Tg(*Runx1*:*FLT3*^ITD^*IDH2*^R172K^): 221.5 days; Tg(*Runx1*:*FLT3*^ITD^*IDH2*^R140Q^): 240 days] (Fig. 2K). The surviving fish also had significant weight loss as they became more mature (Fig. S6I), as compared with their siblings with single or no mutation.

### Transplantability of *IDH2*^R140Q^ and *IDH2*^R172K^ AML cells

**Fig. 3:**
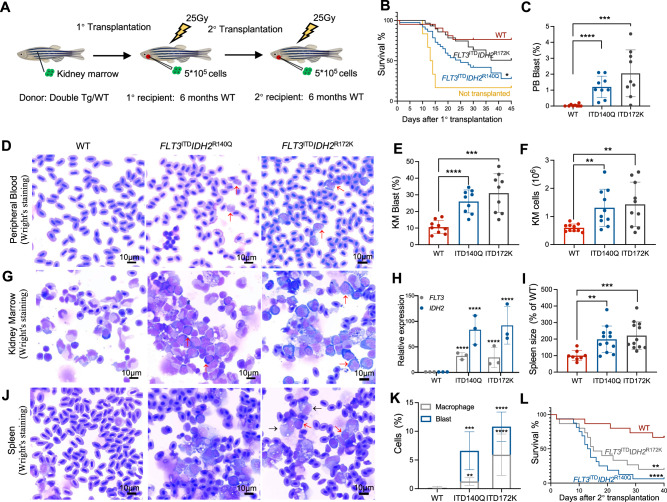
Expanded leukemic blast population and transplantability of *FLT3*^ITD^ and *IDH2* double mutant zebrafish. **A** Schematic representation of the transplantation procedure. **B** Survival plots of the irradiated recipients without transplantation (*n* = 6) or transplanted with KM cells from double transgenic mutant zebrafish (*n* = 32 for *FLT3*^ITD^*IDH2*^R140Q^, *n* = 25 for *FLT3*^ITD^*IDH2*^R172K^) or WT(*n* = 21) 40 days post-primary transplantation. **C** The percentage of the blast cell in the PB and the representative Wright’s staining of the PB (**D**) of the primary recipients (*n* = 6 for all groups) 30 days post-transplantation. **E** The percentage of the blast cell and the total cell number (**F**) in the KM and the representative Wright’s staining of the KM (**G**) of the primary recipients (*n* = 6 for all groups) 30 days post-transplantation. **H** Relative mRNA expression of *FLT3* and *IDH2* in the KM of the primary recipients (*n* = 3 for all groups). **I** The size and the representative Wright’s staining of the spleen (**J**), and the percentage of the blast cell and macrophages in the spleens (**K**) of the primary recipients (*n* = 5 for all groups) 30 days post-transplantation. Blast cells and macrophages are indicated by red and black arrows respectively. **L** Survival plot of the irradiated secondary recipients (*n* = 16 for *FLT3*^ITD^*IDH2*^R140Q^, *n* = 15 for others) 40 days post-transplantation. Data are mean ± s.e.m. Student’s *t* test was performed for **C**, **E**, **F**, **H**, **I**, and **K** (each mutant group vs. WT), **P < 0.01, ***P < 0.001, ****P < 0.0001. Log-Rank test was performed for **B** and **L**, *P < 0.05, **P < 0.01, ****P < 0.0001.

To determine the oncogenic potential of *IDH2*^R140Q^ and *IDH2*^R172K^ AML in zebrafish, their abilities to propagate upon transplantation were examined using transgenic fish of Tg(*Runx1*:*FLT3*^ITD^) background (Fig. 3A). Lethally irradiated WT adult fish could be rescued by WT KM (Fig. 3B). Tg(*Runx1*:*FLT3*^ITD^*IDH2*^R140Q^) and Tg(*Runx1*:*FLT3*^ITD^*IDH2*^R172K^) recipients showed a significantly shorter survival [Median survival: WT: not reach; Tg(*Runx1*:*FLT3*^ITD^*IDH2*^R172K^): not reach; Tg(*Runx1*:*FLT3*^ITD^*IDH2*^R140Q^): 25 days; Not transplanted: 13.5 days] (Fig. 3B). Successful engraftment by leukemic clones was shown by the increase in blasts in the blood (Fig. 3C, D) and KM (Fig. 3E, G) and an increase in cellularity (Fig. 3F) of the latter 30 days post-transplantation. The recipient marrow showed expression of human *FLT3* and *IDH2*, which were not present in the recipients of wildtype marrow (Fig. 3H). Spleen size (Fig. 3I) in the recipients has increased and there were also increases in macrophages and blasts in the spleen of recipients transplanted with Tg(*Runx1*:*FLT3*^ITD^*IDH2*^R172K^) and Tg(*Runx1*: *FLT3*^ITD^*IDH2*^R140Q^) KM (Fig. 3J, K). Secondary transplantation was also performed. Leukemic engraftment was significantly more aggressive [Median survival: WT: not reach; Tg(*Runx1*:*FLT3*^ITD^*IDH2*^R172K^): 15 days; Tg(*Runx1*:*FLT3*^ITD^*IDH2*^R140Q^): 13.5 days] (Fig. 3L).

### Co-expression of *FLT3*^ITD^ and *IDH2* mutation promoted leukemogenesis at the transcriptional level

**Fig. 4:**
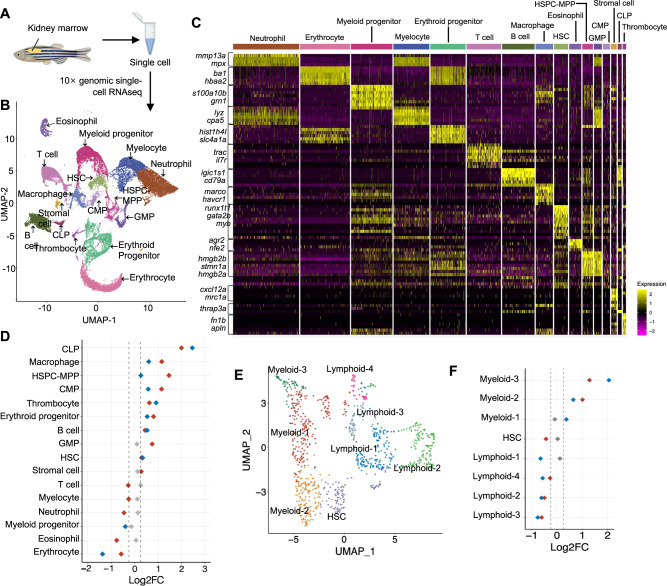
Combined expression *FLT3*^ITD^ and *IDH2* mutations initiated myeloid skewing at the HSPC level in zebrafish. **A** Schematic representation of the single-cell RNA sequencing experimental workflow. **B** 16 distinct cell clusters were visualized by Uniform manifold approximation projection (UMAP). **C** Cell type classification based on the key hematopoietic markers identified in zebrafish. **D** Quantitation of proportions of distribution between double mutant and WT KM cells in different clusters. **E** Sub-clustering of HSPC-MPP population. **F** Quantitation of proportions of distribution between double mutant and WT zebrafish in the different sub-clusters of HSPC-MPP. The red dot represents the difference of the Log2FC of the proportion of a specific cluster in Tg(*Runx1:FLT3*^ITD^*IDH2*^R140Q^) compared to that of WT is over 0.25 or less than −0.25, the blue dot represents the difference of the Log2FC of the proportion of a specific cluster in Tg(*Runx1:FLT3*^ITD^*IDH2*^R172K^) compared to that of WT is over 0.25 or less than −0.25, and the gray dot indicates no change (−0.25 < log2FC < 0.25) was found in the proportion of a specific cluster when compared between the double mutant and WT zebrafish.

To investigate the cellular compartments in Tg(*Runx1*:*FLT3*^ITD^*IDH2*^R172K^) and Tg(*Runx1*:*FLT3*^ITD^*IDH2*^R140Q^) transgenic zebrafish, single-cell RNA-sequencing was performed (Fig. 4A). After removing doublets and filtering by sequencing quality control, 34,214 cells were included in subsequent analyses. Transcriptomes from WT and transgenic mutant KM cells were pooled and clustered using R Seurat Package, resulting in 16 major cell populations (Fig. 4B, C), which were identified by previously reported lineage-specific marker genes [18–28] (Fig. S7A) and were visualized using the Uniform Manifold Approximation and Projection (UMAP) approach (https://arxiv.org/abs/1802.03426) (Fig. 4B). KM from both transgenic fish showed an increase in the prevalence of CLP, macrophage, HSPC-MPP, CMP, thrombocyte, erythroid progenitor, B-cell and HSC populations but a decrease in erythrocyte population when compared with WT siblings (Fig. 4D), indicating multi-lineage hematopoietic expansion by combined expression of *IDH2* mutations and *FLT3*^ITD^ when they were expressed in HSPC as driven by the *Runx1* enhancer and mouse β-globin minimal promoter.

To investigate the mechanisms of myeloid leukemogenesis in the transgenic fish, the HSPC-MPP populations were further examined for possible lineage skewing. Eight sub-clusters were identified, including three myeloid subclusters with up-regulation of myeloid gene *s100a10b*, one HSC subcluster characterized by *myb* expression and four lymphoid subclusters with increased expression of *igic1s1* (Fig. 4E and Fig. S7B). Both mutant *IDH2* transgenic fish showed a significant increase in the prevalence of myeloid subclusters and a significant decrease of lymphoid subclusters (Fig. 4F), suggestive of HSPC-MPP priming towards a myeloid fate.

**Fig. 5:**
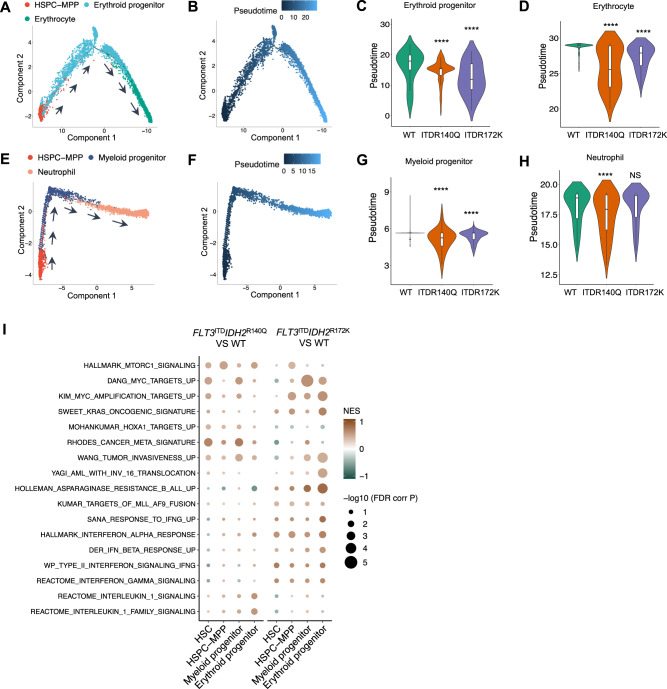
Single-cell RNA sequencing revealed differentiation blockade and enrichment of leukemia-associated gene signatures in *FLT3*^ITD^ and *IDH2* double mutant zebrafish. **A** Lineage differentiation trajectory and pseudotime (**B**) analysis for the erythroid lineage, with HSPC-MPP as the starting point. **C** Quantification of the pseudotime values of the erythroid progenitor and erythrocyte (**D**) in the double mutant and wildtype zebrafish. **E** Lineage differentiation trajectory and pseudotime (**F**) analysis for the myeloid lineage, with HSPC-MPP as the starting point. **G** Quantification of the pseudotime values of the myeloid progenitor and neutrophil (**H**) in the double mutant and WT zebrafish. **I** Differentially enriched Hallmark and C2 pathway signatures within the HSC, HSPC-MPP, myeloid, and erythroid progenitor clusters between Tg(*Runx1:FLT3*^ITD^*IDH2*^R140Q^) and WT or Tg(*Runx1:FLT3*^ITD^*IDH2*^R172K^) and WT. Wilcoxon Rank Sum test was performed for **C**, **D**, **G**, and **H**, ****P < 0.0001.

To examine the effects of *IDH2* mutations on lineage differentiation, lineage trajectory and pseudotime analyses were performed for erythroid and myeloid lineages (Fig. 5A–H). Lineage-specific progenitors and terminally differentiated mature cells were differentiated from HSPC-MPP, with increasing pseudotime values along differentiation (Fig. 5A, B, E and F). Pseudotime of lineage-specific progenitors and erythrocytes from Tg(*Runx1*:*FLT3*^ITD^*IDH2*^R140Q^) and Tg(*Runx1*:*FLT3*^ITD^*IDH2*^R172K^), and that of neutrophils from Tg(*Runx1*:*FLT3*^ITD^*IDH2*^R140Q^), were significantly lower than those of their WT siblings (Fig. 5C, D, G and H). Interestingly, while these mutation combinations induced myeloid priming of HSPC-MPP, as shown by a significant increase in the prevalence of myeloid subclusters and significant decrease of lymphoid subclusters within this population, they also induced differentiation blockage downstream of HSPC-MPP, as shown by the lower pseudotime in the downstream populations.

To further evaluate the effects of *IDH2* mutations on the initiation and promotion of leukemogenesis, gene set enrichment analysis (GSEA) was performed for early hematopoietic cell populations, including HSC, HSPC, myeloid, and erythroid progenitor cell clusters, based on differentially expressed genes between Tg(*Runx1*:*FLT3*^ITD^*IDH2*^R172K^), Tg(*Runx1*:*FLT3*^ITD^*IDH2*^R140Q^) and their WT siblings (Fig. 5I). In both double transgenic fish, genes associated with MTORC, MYC and RAS signaling, were positively enriched in most cell clusters, consistent with their leukemia phenotypes. Intriguingly, the Tg(*Runx1*:*FLT3*^ITD^*IDH2*^R172K^) zebrafish showed positive enrichment of genes associated with interferon responses and signaling whereas the Tg(*Runx1*:*FLT3*^ITD^*IDH2*^R140Q^) zebrafish showed positive enrichment of genes associated with interleukin 1-related signaling.

### Use of transgenic fish in therapeutic evaluation

**Fig. 6:**
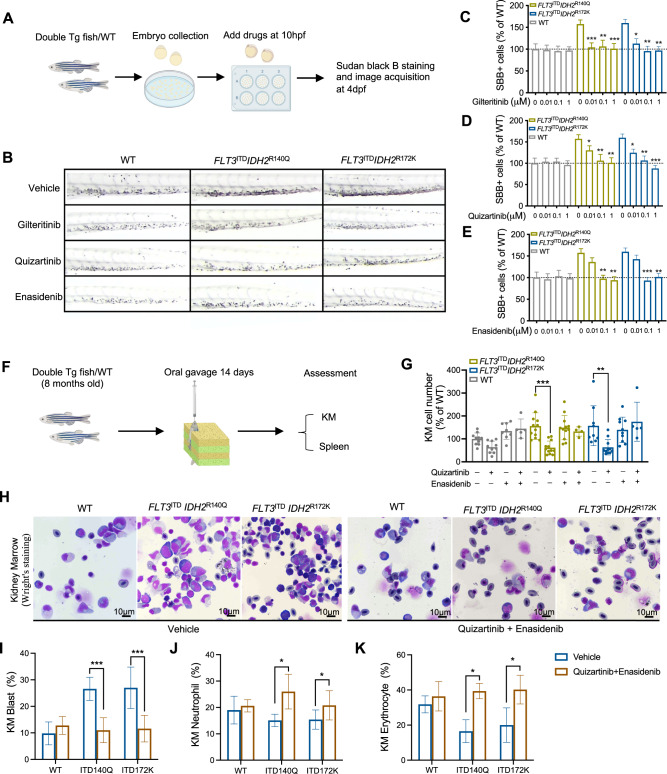
Response of the *FLT3*^ITD^ and *IDH2* double mutant zebrafish to therapeutic treatments. **A** Schematic representation of drug administration and assessment in zebrafish embryos. **B** Representative SBB staining in the double mutant and WT embryos following in vivo treatment of vehicle control, gilteritinib, quizartinib, and enasidenib at 0.01 μM, 0.1 μM and 1 μM respectively. **C–E** Quantification of SBB + cells, presented as the percentage of SBB + cells of treated zebrafish for relative to untreated WT control (*n* = 20 for all groups) in **B**. **F** Schematic representation of drug administration and assessment in adult zebrafish. **G** Changes in total KM cellularity in the adult double mutant and WT zebrafish post-treatment. **H** Representative Wright’s staining of the KM from the adult double mutant and WT zebrafish 14 days post-treatment. **I** The percentage of blast cell, neutrophil (**J**), and erythrocyte (**K**) in the KM of double mutant and WT zebrafish (*n* = 5 for all groups). Data are mean ± s.e.m and statistical analysis was performed by Student’s *t* test (treated vs. untreated for each genotype), *P < 0.05, **P < 0.01, ***P < 0.001.

The clinical relevance of the zebrafish models was tested at both embryonic and adult stages. Tg(*Runx1:FLT3*^ITD^*IDH2*^R172K^) and Tg(*Runx1:FLT3*^ITD^*IDH2*^R140Q^) embryos and their WT siblings were treated with gilteritinib and quizartinib (FLT3 inhibitors) as well as enasidenib (IDH2 inhibitor), which have been shown to induce clinical response and confer a survival advantage to patients with *FLT3*^*ITD*^ and *IDH2* mutations (Fig. 6A). Using cytochemical staining with SBB as a surrogate for embryonic myelopoiesis, these inhibitors ameliorated the increase in myelopoiesis in the double transgenic embryos (Fig. 6B–E). The therapeutic responses were also tested in adult fish (Fig. 6F). Initial dose-finding studies showed that daily gavage of quizartinib at 10 mg/kg and enasidenib at 100 mg/kg were compatible with normal fish survival (Fig. S8A, B). Double transgenic leukemic fish and their WT siblings were treated with 14 days of quizartinib, enasidenib, or their combination, and their KM cellularity, blast, neutrophil, and erythrocyte counts were enumerated (Fig. 6G–K). Quizartinib but not enasidenib monotherapy significantly reduced cellularity in the KM of the double transgenic fish (Fig. 6G). Enasidenib but not quizartinb monotherapy significantly increased the percentage of neutrophil and erythrocyte and slightly decreased the blast cell population in the KM of the double transgenic fish (Fig. S8C–I). Combination treatment significantly reduced blast population (Fig. 6H, I), increased neutrophil abundance (Fig. 6H, J), and restored erythropoiesis in the KM (Fig. 6H, K). When the effects of these therapeutic agents on the spleen were examined, only the combination of quizartinib and enasidenib effectively reduced the size of the spleen (Fig S8J, K).

## Discussion

*IDH2*^R172K^ and *IDH2*^R140Q^ mutations occur in more than 15% of patients with cytogenetically normal AML. Despite their frequent occurrence, comparative studies of their roles in leukemogenesis have been scarce. In this study, we generated transgenic zebrafish models and demonstrated that transgenic expression of *IDH2*^R172K^ and *IDH2*^R140Q^ in hematopoietic stem/progenitor cells induced myeloid skewing and differentiation blockade at HSPC-MPP levels, resulting in expansion of KM, splenomegaly, myelodysplasia and increase in Mpo+ blasts capable of self-renewal in serial transplantations. The leukemic phenotypes responded to target therapies expected of their mechanisms of action, attesting to the clinical relevance of the zebrafish models and their potential application in the development of personalized medicine. These findings were consistent with mouse models of transgenic or knockin *IDH2*^R140Q^ [7, 9, 29, 30], where *IDH2* mutation was shown to induce differentiation block and leukemogenesis, either singly or in combination with other genetic perturbations. Moreover, observations arising from this study may shed important lights to our understanding of leukemogenesis pertinent to *IDH2* mutation.

First, our observations of *IDH2*^R172K^ AML in zebrafish were consistent with those in viral transduction studies where *IDH2*^R172K^ expression in hematopoietic cells induced leukemogenesis [6, 7] but was different from the knock-in mouse model in which *IDH2*^R172K^ expression in hematopoietic cells led to perturbed lymphoid development but not leukemia [10, 11]. Different experimental models and tissue promoters chosen for *IDH2*^R172K^ expression as well as cooperative mutation partners might account for the difference in phenotypes. In zebrafish, *IDH2*^R172K^ alone was found to induce leukemia-like phenotypes in a small percentage of the transgenic zebrafish, and full-blown leukemia was developed in those fish with *IDH2*^R172K^ in the combination of *FLT3*^ITD^. Their cooperativity was also shown by the superior pharmacologic responses to the combination of enasidenib and quizartinib, inhibitors of *IDH2*^R172K^ and *FLT3*^ITD^, underscoring the pathogenetic role of each mutant gene in this model.

Second, we demonstrated that in addition to *IDH2*^R140Q^, transgenic expression of *IDH2*^R172K^ in HSPC induced differentiation blockade of hematopoiesis, as illustrated by trajectory analyses of the single-cell transcriptome. The lower pseudotime of lineage-specific progenitors and mature neutrophils and erythrocytes as they differentiated from HSPC-MPP supported the proposition of differentiation blockade in both *IDH2* mutant AML. Furthermore, the rapid recovery of neutrophils and erythrocytes upon treatment with enasidenib and its combination with quizartinib also suggested a relief of differentiation blockade reminiscent of patient response to enasidenib.

Third, single-cell transcriptome analysis of the KM has empowered us to examine the lineage development and hematopoietic phenotypes of zebrafish AML in detail. Both Tg(*Runx1:FLT3*^ITD^*IDH2*^R172K^) and Tg(*Runx1:FLT3*^ITD^*IDH2*^R140Q^) showed multi-lineage hematopoietic expansion in KM as compared with wildtype siblings. Expression of these transgenes in HSPC as driven by the *Runx1* enhancer and mouse β-globin minimal promoter could lead to clonal expansion of both lymphoid and myeloid progenitors, notably CLP and CMP. A close examination of HSPC-MPP showed full-blown lineage skewing typical of myeloid neoplasm, suggesting leukemogenesis might begin at the HSPC-MPP stage in the zebrafish model. Moreover, GSEA analysis revealed potential associations between Tg(*Runx1:FLT3*^ITD^*IDH2*^R172K^), Tg(*Runx1:FLT3*^ITD^*IDH2*^R140Q^) and immune activation with particular reference to interferon and IL-1 signal activation. Their mechanistic links would have to be further evaluated.

Finally, information arising from this study has shed important lights to the hitherto undescribed hematopoietic effects of *IDH2* mutations. In particular, *IDH2*^R172K^ but not *IDH2*^R140Q^, accentuated T-cell development at both embryonic and adult stages. The effects were cell-autonomous and *IDH2*^R172K^ mutation could be demonstrated in the thymus. It was unclear if the preferential effects of *IDH2*^R172K^ on T-cell development were related to a significantly higher level of oncometabolite 2HG, as demonstrated in zebrafish embryos and mice [10]. To our knowledge, *IDH2*^R172K^ has not been reported in precursor T-cell lymphoblastic leukemia. Whether the *IDH2*^R172K^ transgenic zebrafish would provide a disease model of mature T-cell neoplasm, including angioimmunoblastic T-cell lymphoma, in which *IDH2*^R172K^ mutation occurs in 20% of cases [31–33], would have to be further investigated [10].

The zebrafish model was of clinical relevance. As proof-of-principle, both zebrafish embryos and adults carrying *FLT3*^ITD^*IDH2*^R172K^ and *FLT3*^ITD^*IDH2*^R140Q^ mutations responded to quizartinib/gilteritinib, and enasidenib, which were effective agents for *FLT3*^ITD^ [34, 35] and *IDH2* mutant AML [36, 37]. The optical transparency, high fecundity and relatively simple husbandry of zebrafish have made it uniquely suitable for high throughput drug screening. Furthermore, the availability of lineage-specific fluorescent reporter lines has also facilitated the evaluation of drug effects on specific lineages. Intriguingly, increased HSPC and enhanced primitive myelopoiesis were observed only in monogenic *IDH2*^R172K^ and *IDH2*^R140Q^ expression in the transient but not stable transgenic model. The apparent discrepancy might be explained by a ubiquitous and higher level of mutant gene expression in the transient system. Whether the transient expression system might provide a more robust high throughput model for zebrafish drug screening would have to be further tested.

In conclusion, the present study generated zebrafish models of AML carrying *FLT3*^ITD^*IDH2*^R172K^ and *FLT3*^ITD^*IDH2*^R140Q^ mutations, recapitulating the morphologic, clinical, transcriptomic and functional characteristics of the corresponding human diseases. These double transgenic fish will become prototypes of zebrafish AML models carrying mutation combinations and powerful tools for rapid drug discovery targeting specific drive mutations.

## Methods

### Generation of transgenic zebrafish lines

Tol2 transgenesis was used to generate single transgenic fish lines Tg(*Runx1: IDH2*^R140Q^) *and* Tg(*Runx1: IDH2*^R172K^) in which human *IDH2*^R140Q^ or *IDH2*^R172K^ were expressed under the control of the HSPC-specific *Runx1* + 23 enhancer and mouse β-globin minimal promoter (Fig. S1A) [38]. Human sequence of *IDH2*^R140Q^ or *IDH2*^R172K^ was cloned into pDONR221 vector to generate the “middle” clone (pME*-IDH2* ^R140Q^ or *IDH2* ^R172K^) by Gateway BP reaction. The final Tol2 integrable construct was generated via multisite Gateway LR reaction in which three entry clones [p5E‐*Runx1* + 23 (Addgene #69602), pME‐ *IDH2*^R140Q^ or *IDH2*^R172K^, and p3E‐mCherrypA] [39] were incorporated into the destination vector pDestTol2CG [39] with EGFP fluorescent protein expressed under the control of the cardiomyocyte-specific *cmlc2* promoter. The latter served as a marker of successful transgenesis. Single transgenic lines were generated by co-injecting 50–100 pg of the respective Tol2 construct and the Tol2 transposase mRNA into the wildtype (WT) TU embryos at the one-cell stage. Founders were identified by PCR and EGFP fluorescence of the heart. F1 single transgenic fish were generated by outcrossing the identified founder fish with WT TU fish. F1 embryos with positive heart EGFP fluorescence at 2dpf were raised to adulthood (Fig. S1B), and the genotype was further confirmed by genotyping (Fig. S1C). The generation of Tg(*Runx1:FLT3*^ITD^) transgenic line was described previously [17]. Double transgenic fish Tg(*Runx1:FLT3*^ITD^*IDH2*^R140Q^) and Tg(*Runx1:FLT3*^ITD^*IDH2*^R172K^) were generated by crossing the single transgenic lines of Tg(*Runx1: IDH2*^R140Q/R172K^) with Tg(*Runx1:FLT3*^ITD^). The expression of *FLT3* and *IDH2* mRNA in F1 adult fish was confirmed by q-PCR(Fig. S1D). F1 transgenic fish were also outcrossed with different transgenic reporter lines, including Tg(*rag2*:EGFP) and Tg(*mpo*: EGFP) for evaluation of thymus and kidney marrow size.

### Zebrafish kidney marrow transplantation

WT recipient fish were irradiated at 25 Gy 2 days prior to transplantation. On the day of transplantation, donor KM cells were collected from Tg(*Runx1: FLT3*^ITD^*IDH2*^R172K^), Tg(*Runx1: FLT3*^ITD^*IDH2*^R140Q^) or WT fish in 0.9X PBS with 5% FBS buffer, resuspended in injection medium (0.9X PBS, 5% FBS, 0.5 U/μL heparin and 0.2 U/μL Dnase I) to a final concentration of 2 × 10^5^ cells/μL [40] Recipient fish was anesthetized using 0.015% Tricaine and 5 × 10^5^ donor cells were transplanted via intracardiac injection using capillary glass needle (GC100TF-15, Warner Instruments, USA). After injection, recipients were immediately returned to sterilized fish water. Survival of recipients was monitored and recorded daily.

### Single-cell RNA sequencing

Sample preparation, library preparation, sequencing, and data analysis details are provided in the Supplementary materials and methods. A total of 34,214 KM cells passed the quality control and were analyzed using Seurat (v4.0.6) [41]. FindClusters were used to determine cell clusters. Marker genes for each cluster were inspected and cluster identities were determined based on previously reported lineage-specific marker genes in zebrafish [18–28]. Differentially expressed genes were analyzed based on their known functions according to the curated hallmark and C2 gene sets from Molecular Signatures Database (MSigDB v7.4 [42, 43] https://www.gsea-msigdb.org/gsea/msigdb/index.jsp). Lineage trajectory and pseudotime analysis were performed by Monocle 2 [44, 45].

### Drug treatment on embryos and adult fish

Tg*(Runx1:FLT3*^ITD^*IDH2*^R172K^), Tg*(Runx1:FLT3*^ITD^*IDH2*^R140Q^) and WT zebrafish embryos were treated with FLT3 inhibitors Gilteritinib (Selleck S7754), Quizartinib (MedChemExpress HY-13001), or IDH2 inhibitor Enasidenib (MedChemExpress HY-18690) at three concentrations (0.01, 0.1, and 1 μM) in E3 medium with 1-phenyl-2-thiourea at 6 hpf. The medium was changed every 24 h. At 4 dpf, embryos were collected and fixed in 4%PFA for SBB staining. Adult zebrafish were anesthetized using 0.015% Tricaine, propped vertically in a damp sponge, and 5–10 μl of quizartinib (10 mg/kg), enasidenib (100 mg/kg) or their combination, dissolved in H_2_O containing 10% DMSO (Sigma) and 0.01% Phenol Red (Sigma-Aldrich), were given via oral gavage [46, 47]. Treated zebrafish were placed into individual recovery tank with fresh and sterile fish water immediately. Treatment was given daily for 14 days.

### Statistical analysis

Data were assessed for normal distribution with a Shapiro–Wilk normality test using Prism9 (GraphPad, San Diego, CA) and presented as mean ± standard error of the mean (s.e.m) of at least three independent experiments. Sample sizes were determined by power analysis to provide sufficient statistical power to detect differences. Comparisons between group of data were evaluated by two-sided Student’s *t* test, one-way ANOVA, or Wilcoxon Rank Sum test if the data were normally distributed and the variance was equal. Survival data were evaluated by Kaplan-Meier analyses and compared using Log-Rank test. *P* value less than 0.05 was considered statistically significant.

Additional methods and materials used in this study are provided in the Supplementary Information.

## Supplementary information

Supplementary methods

Supplementary figures

## Data Availability

Single-cell RNA sequencing data have been deposited in the National Center for Biotechnology Information Sequence Read Archive under accession number PRJNA911847.
