# Correction: Transgenic *IDH2*^R172K^ and *IDH2*^R140Q^ zebrafish models recapitulated features of human acute myeloid leukemia

**DOI:** 10.1038/s41388-023-02664-z

**Published:** 2023-03-15

**Authors:** Dandan Wang, Lichuan Zheng, Bowie Yik Ling Cheng, Chun-Fung Sin, Runsheng Li, Sze Pui Tsui, Xinyu Yi, Alvin Chun Hang Ma, Bai Liang He, Anskar Yu Hung Leung, Xuan Sun

**Affiliations:** 1 Division of Haematology, Department of Medicine, LKS Faculty of Medicine, The University of Hong Kong, Pokfulam, Hong Kong SAR, China; 2 Department of Pathology, Queen Mary Hospital, Hong Kong SAR, China; 3 Department of Infectious Diseases and Public Health, City University of Hong Kong, Hong Kong SAR, China; 4 BGI-Genomics, BGI-Shenzhen, Shenzhen, China; 5 Department of Health Technology and Informatics, The Hong Kong Polytechnic University, Hung Hom, Hong Kong SAR, China; 6 Guangdong Provincial Key Laboratory of Biomedical Imaging and Guangdong Provincial Engineering Research Center of Molecular Imaging, The Fifth Affiliated Hospital, Sun Yat-sen University, Zhuhai, Guangdong 519000 China

**Keywords:** Acute myeloid leukaemia, Cancer genomics, Sequencing

Correction to: *Oncogene* 10.1038/s41388-023-02611-y, published online 04 February 2023

The figure legends for Figs. 1, 2, 3, 6 and Supplementary Figs. 3, 4, 6, 8 should read *P < 0.05 instead of *P < 0.5.

The original article has been corrected.

