# Supplementary material for: Transgenic IDH2R172K and IDH2R140Q zebrafish models recapitulated features of human acute myeloid leukemia
Source: Oncogene. 2023 Feb 4;42(16):1272–81. doi: 10.1038/s41388-023-02611-y (PMC10101851; doi:10.1038/s41388-023-02611-y)
Supplement: Supplementary file 1 — Supplementary methods [file 41388_2023_2611_MOESM1_ESM.docx]

**Supplementary Information**

**Zebrafish husbandry**

Wildtype Tubingen (TU), Tg(*rag2*: EGFP) and Tg(*mpo*: EGFP) zebrafish lines were purchased from Zebrafish International Resource Center (ZIRC, USA). The study was approved by the Committee of the Use of Laboratory and Research Animals (CULATR) at the University of Hong Kong (HKU). An assessor blinded to zebrafish genotype performed the initial analysis and data collection. Details of zebrafish husbandry and protocols of whole-mount in-situ hybridization (WISH) and quantitative RT-PCR were described previously (1-3). Primer sequences for genotyping and RT-PCR were listed in Supplementary Table 1.

**Generation of stable transgenic zebrafish lines expressing human *IDH2* mutations**

Transgenic zebrafish expressing human *IDH2*^R172K^ [Tg(*Runx1*:*IDH2*^R172K^)] in hematopoietic stem and progenitor cells, driven by *Runx1* enhancer and mouse β-globin minimal promoter, were generated using Tol2 transgenesis (Fig. S1A). Transgenic Tg(*Runx1*:*IDH2*^R140Q^) zebrafish was similarly generated for comparation. Successful transgenesis was shown by EGFP (driven by *cmlc2*) expression in the developing heart (Fig. S1B) and confirmed by PCR genotyping (Fig. S1C). Adult zebrafish were outcrossed with WT (TU) to confirm germline transmission to F1. These lines were crossed to Tg(*Runx1*:*FLT3*^ITD^) background (1) to generate Tg(*Runx1*:*FLT3*^ITD^*IDH2*^R140Q^) and Tg(*Runx1*:*FLT3*^ITD^*IDH2*^R172K^). Fish were assigned to different groups according to their genotype. The expression of human *FLT3* and *IDH2* in KM of the mutant fish was confirmed via qPCR (Fig. S1D).

**Sudan Black B (SBB) staining**

Zebrafish embryos were collected and fixed in 4% Paraformaldehyde (PFA) overnight at 4°C, followed by repeated washing with Phosphate-Buffered Saline solution with 1% Tween ™ 20 (PBST). They were then incubated in Sudan Black B Staining Reagent diluted 1:10 in 70% ethanol for 30 minutes at room temperature. Afterward, embryos were washed extensively in 70% ethanol, then serially rehydrated with decreasing ethanol concentration in PBST, eventually in PBST only. Embryos were then mounted in 3% methylcellulose and imaged with CWHC-1080B 4K cMOS camera (Chinetek Scientific, China) attached to a Nikon SMZ800 stereomicroscope (Nikon, Japan) and analyzed using Fiji (4).

**Kidney marrow (KM) collection for histologic analysis**

Zebrafish KM were harvested from 8 months old transgenic or WT fish, dissociated in 0.9X PBS with 5% fetal bovine serum (FBS) by pipetting, and filtered through a 40 µm nylon cell strainer (Corning, NY, USA). The cells were centrifuged at 500g for 5 minutes, resuspended, filtered and washed with 0.9X PBS with 5% fetal bovine serum (FBS) at least twice. Cytospin preparations were made with 2x10^5^ to 3x10^5^ cells cytocentrifuge (epredia, USA) at 400 RPM for 5 minutes onto the microscope glass slides. The slides were dried at room temperature for more than 12 hours, stained for Wright’s stain or myeloperoxidase (Mpo), and imaged with Nikon DS-Ri2 digital camera (Nikon, Japan) fitted to a Nikon Eclipse Ni-U microscope (Nikon, Japan).

**Blood collection and blood smear**

Glass capillary (GC100TF-15, Warner Instruments, USA) was soaked in 5mg/ml heparin and air-dried at room temperature for at least 1 hour. Adult fish were anesthetized with 0.015% Tricaine in E3 medium and placed onto a dissection board covered with wet paper towels. The blood collection apparatus comprised a heparinized needle, a silicone tubing (0.8mm ID, Biorad) and a DistriTip microsyringe (125μl, Gilson). Blood was collected by inserting the glass needle at a 45°-90° angle into the dorsal aorta (5) of the zebrafish and 5-10 ul of blood could be drawn at each time to prepare for a blood smear. The slides of the blood smear were stained for Wright’s. Cellular morphology was imaged with Nikon DS-Ri2 digital camera fitted to a Nikon Eclipse Ni-U microscope (Nikon, Japan).

***In Vivo* Microscopy**

Adult fish with different reporter backgrounds, including Tg(*mpo*: EGFP) and Tg(*rag2*: EGFP) were anesthetized with 0.015% Tricaine in E3 medium. Whole animal images were taken with a Carl Zeiss AxioZoom.V16 microscope and Axiocam 208 color camera (Zeiss, Germany). Images were captured using ZEN 3.2 Blue Edition and analyzed using Fiji (4).

**Measurement of 2HG via Gas chromatography – Mass Spectrometer (GC-MS)**

The level of 2HG in embryos were measured via GC-MS at the Proteomics and Metabolomics Core of the University of Hong Kong. Protein lysate was extracted from the embryos and processed for GC-MS analysis. GC-MS chromatogram was acquired in SCAN and MRM mode using an Agilent 7890B GC - Agilent 7010 Triple Quadrapole Mass Spectrometer system (Santa Clara, CA, USA). Data analysis was performed using the Agilent MassHunter Workstation Quantitative Analysis Software (Agilent Technologies, USA). Linear calibration curves for each analyte were generated by plotting the peak area ratio of external/internal standard against standard concentration at different concentration levels. Analytes were confirmed by comparing the retention time and ratio of characteristic transitions between samples and standards.

**Sample preparation, library construction and single-cell RNA sequencing**

Single viable KM cells were collected from the transgenic double mutant (n=3; pooled) and WT (n=3; pooled) fish at 7 months old into 0.9X PBS with 5% FBS. Their viability was examined by 0.4% Trypan blue staining under microscopy. The single-cell library was constructed using the Chromium^TM^ Controller and Chromium^TM^ Next GEM Single Cell 3’ Kit v3.1 (10x Genomics, Pleasanton, CA). Complementary DNA (cDNA) was synthesized from the fragmentated RNAs using N6 random primers, followed by end repair and ligation to BGISEQ sequencer compatible adapters. Quality control of the final library was performed by checking the distribution of the fragments size using the Agilent 2100 bioanalyzer and quantification was performed by real-time quantitative PCR using TaqMan probes. The final products were sequenced using the DNBSEQ^TM^ platform (BGI- HK, China).

Raw sequencing output was processed by Cell Ranger (v3.1.0) to generate gene count matrix. Data was served as input for Seurat (v4.0.6), and cells with unique molecular identifier (UMI) < 500 or > 60000 were filtered out. Cells with mitochondrial genes ratio > 10% were discarded. scDoubletFinder was used for identification of potential doublets based on the default parameter. After filtering, 34214 cells passed the quality control and were processed in downstream analysis. Cells passing all steps of quality control were processed in Seurat. The raw count matrix was normalized using the NormalizeData function. Top 3000 variable genes were identified and the count matrix was scaled with FindVariableFeatures and ScaleData functions, respectively. Top 30 principle components were calculated using RunPCA function. Batch correction and integration were performed based on mutual nearest neighbors (MNN) between datasets using canonical correlation analysis (CCA). After fitting data into an integrated space, Louvain algorithm was utilized to optimize and determine the cell clusters based on FindClusters. Differentially expressed genes (DEGs) were computed for each cluster by a hurdle model implemented as MAST algorithm with log-normalized counts matrix. Gene set enrichment analysis (GSEA) was performed using clusterProfiler R package (6), and the result was visualized by ggplot2 (7).

To evaluate the differentiation process, lineage trajectory was inferred by Monocle 2 (8, 9). Briefly, differentiation paths were extracted from the major cell clusters of the erythroid and myeloid lineages. Top 500 differentially expressed genes were identified using differentialGeneTest function. Dimensional reduction was performed based on ‘DDRTree’ method with default parameter, after which pseudotime was assigned using orderCells function with ‘HSPC-MPP’ cells selected as the root node. Pseudotime for each cell type between the transgenic double mutant and WT zebrafish were compared.

**Legends to Supplementary Figures**

**Fig. S1. Generation of stable transgenic zebrafish lines with *IDH2* mutations.** **A**, Schematic diagrams of the Tol2 constructs of *Runx1*: *IDH2*^R140Q/172K^. **B**, Successful integration of the Tol2 construct was indicated by cardiac EGFP fluorescence signal in embryos at 24hpf and 48hpf. **C**, The presence of *IDH2* mutation in transgenic zebrafish was confirmed via genotyping. **D**, Expression of *FLT3* and *IDH2* mRNA in the KM of the stable mutant zebrafish (n=3 for all groups) were confirmed via q-PCR, and the relative expression of each transgene was represented as its expression in mutant zebrafish relative to the wildtype (WT) control. Data are mean ± s.e.m and statistical analysis was performed by Student’s t-test (each mutant group vs. WT), ****P<0.0001.

**Fig. S2. Comparison between human *IDH2* vs. zebrafish *idh2*. A**, Syntenic neighboring genes (SNGs) of *IDH2* in humans and zebrafish. **B**, Protein sequence alignment of and IDH2/Idh2 between humans and zebrafish.

**Fig. S3. Transient effects of *IDH2* mutations on embryonic hematopoiesis in zebrafish. A.** The level of 2HG in embryos microinjected with *IDH2* mutant mRNA and uninjected WT embryos**. B**, Brightfield images of WISH for *pu.1* at 24hpf, *cmyb* at 36hpf, *mpo* at 48 hpf and SBB at 48hpf in the embryos microinjected with *IDH2* mutant mRNA (n=20) and uninjected WT embryos (n=20). **C,** Quantification of *pu.1*, *cmyb* **(D)**, *mpo* **(E)** and SBB **(F)**. The expression of each marker was represented as the percentage of each marker expressed in the embryos with *IDH2* mutant mRNA relative to that of the WT embryos. Data are mean ± s.e.m and statistical analysis was performed by Student’s t-test, *P<0.05, ***P<0.001 and ****P<0.0001.

**Fig. S4. Combinatorial effect of *FLT3*^ITD^ and/or *IDH2* mutations on primitive and definitive hematopoiesis in zebrafish embryos. A**, Brightfield images of WISH for *gata1* and *hbae1.1* at 20hpf, *pu.1* at 24hpf, *runx1* at 36hpf, SBB at 48hpf, and *l-plastin* at 48hpf in the embryos of the mutant (n=20) and WT (n=20) zebrafish. **B**, Quantification of *runx1*, SBB (**C**), *l-plastin* (**D**), *gata1* (**E**), *hbae1.1* (**F**) and *pu.1* (**G**). The expression of each marker was represented as the percentage of each marker expressed in the transgenic embryos relative to that of the WT embryos. Data are mean ± s.e.m and statistical analysis was performed by Student’s t-test, *P<0.05, **P<0.01, and ****P<0.0001.

**Fig. S5. Morphology of hematopoietic cells in the kidney marrow of the mutant zebrafish and WT siblings.** Representative Wright’s staining images of different hematopoietic cell types in the KM of the mutant and WT zebrafish.

**Fig. S6. Collaboration of *FLT3*^ITD^ and *IDH2* mutations induced AML-like phenotypes in zebrafish. A**, The size of the KM in the transgenic mutant zebrafish and WT siblings is indicated by white dash lines in Tg(*mpo*:EGFP) background fish. **B**, Representative Wright’s staining of the spleen cells from the transgenic mutant zebrafish and WT siblings. **C**, Relative size of the KM. **D**, Representative images of spleens from the mutant and WT zebrafish. **E**, Relative spleen size and the spleen cellularity (**F**) of the mutant zebrafish (n=10) and WT (n=10) siblings. **G**, The percentage of the myelomonocyte and blast cell in the spleens of the transgenic mutant zebrafish (n=5) and WT siblings (n=5). **H**, Confirmation of the presence of *IDH2* hotspot mutation in the KM and thymus of the mutant zebrafish via Sanger sequencing. **I**, The body weight of the mutant zebrafish (n=8) and WT (n=8) siblings. Data are mean ± s.e.m. One-way Anova was performed for **C**, **E**, **F** and **G**, *P<0.05, **P<0.01, ***P<0.001, ****P<0.0001. Student’s t-test was performed for **I** (each mutant group vs. WT), *P<0.05, **P<0.01.

**Fig. S7. Gene expression profiles of the representative genes in each cell cluster. A**, The expression of top marker gene for different cell clusters. **B**, The expression of *s100a10b*, *myb*, and *igic1s* within the HSPC-MPP cluster.

**Fig. S8. *FLT3*^ITD^ and *IDH2* double mutant zebrafish were sensitive to therapeutic treatments. A**, Survival plots of the WT zebrafish treated with Quizartinib (n=6) or Enasidenib (**B**) (n=6) of 14 days post-treatment. **C**, Representative Wright’s staining of the KM 14 days post-treatment of Quizartinib or Enasidenib. **D**, The percentage of the erythrocyte, neutrophil (**E**), and blast cell (**F**) in the KM 14 days post-treatment of Quizartinib (n=5). **G**, The percentage of the erythrocyte, neutrophil (**H**), and blast cell (**I**) in the KM 14 days post-treatment of Enasidenib (n=5). **J**, Representative images of the spleen post-treatment of Quizartinib and/or Enasidenib. **K**, Quantification of the relative size of the spleen in the double mutant and WT zebrafish post-treatment of Quizartinib and/or Enasidenib. Data are mean ± s.e.m. Log-Rank test was performed for **A** and **B**. Student’s t-test was performed for **D-I** and **K**, *P<0.05, **P<0.01.

**Supplementary Table 1. List of primers.**

| Gene name | Species | Forward primer | Reverse primer |
| --- | --- | --- | --- |
| *FLT3* for genotyping | Human | CTGCCGCTGCTCGTTGTTTT | CCCTCCTCGAGTGCTTTGTT |
| *IDH2* for genotyping | Human | CCAAACCGTGACCAGACTGA | GCCTCAGCCTCAATCGTC |
| *FLT3* for qPCR | Human | TAGAAATCAAGATCTGCCTGAGA | CTGAGCTCTGGGGTCTCAAC |
| *IDH2* for qPCR | Human | CCAAACCGTGACCAGACTGA | CTCATCAGGGGTGATGGTGG |
| *pax5* | zebrafish | CCAACAGGGAGCGGAAGAGTC | GGTTGGACAGAGGGATGAGC |
| *cd79a* | zebrafish | CGGCTCATTTCATACCTAGCAG | AGACCTGCACACGCAGTAAT |
| *cd9a* | zebrafish | CTTCCAGCATGGGTGAATTG | TCATCAATGGCATCAGGGCA |
| *rag1* | zebrafish | TTGTCACAGGTGCTGGACTG | GCTTCCTTCCTACTCGCTGA |
| *cmyb* | zebrafish | TTTCTACCGAATCGAACAGAT | CAATCACCCGTTGGTCTTCT |
| *β-actin* | zebrafish | AATGAGCGTTTCCGTTGCC | CAGGTCCTTACGGATGTCCAC |

**References**

1. He BL, Yang N, Man CH, Ng NK, Cher CY, Leung HC, et al. Follistatin is a novel therapeutic target and biomarker in FLT3/ITD acute myeloid leukemia. EMBO Mol Med. 2020;12(4):e10895.

2. Ma ACH, Shi X, He BL, Guo Y, Leung AYH. A Zebrafish Model for Evaluating the Function of Human Leukemic Gene IDH1 and Its Mutation. Methods Mol Biol. 2017;1633:193-218.

3. Shi X, He BL, Ma AC, Guo Y, Chi Y, Man CH, et al. Functions of idh1 and its mutation in the regulation of developmental hematopoiesis in zebrafish. Blood. 2015;125(19):2974-84.

4. Schindelin J, Arganda-Carreras I, Frise E, Kaynig V, Longair M, Pietzsch T, et al. Fiji: an open-source platform for biological-image analysis. Nature methods. 2012;9(7):676-82.

5. Zang L, Shimada Y, Nishimura Y, Tanaka T, Nishimura N. Repeated Blood Collection for Blood Tests in Adult Zebrafish. J Vis Exp. 2015(102):e53272.

6. Yu G, Wang LG, Han Y, He QY. clusterProfiler: an R package for comparing biological themes among gene clusters. OMICS. 2012;16(5):284-7.

7. Wickham H. ggplot2: elegant graphics for data analysis. springer. 2016.

8. Trapnell C, Cacchiarelli D, Grimsby J, Pokharel P, Li S, Morse M, et al. The dynamics and regulators of cell fate decisions are revealed by pseudotemporal ordering of single cells. Nat Biotechnol. 2014;32(4):381-6.

9. Qiu X, Mao Q, Tang Y, Wang L, Chawla R, Pliner HA, et al. Reversed graph embedding resolves complex single-cell trajectories. Nat Methods. 2017;14(10):979-82.
