# Supplementary material for: Transgenic IDH2R172K and IDH2R140Q zebrafish models recapitulated features of human acute myeloid leukemia
Source: Oncogene. 2023 Feb 4;42(16):1272–81. doi: 10.1038/s41388-023-02611-y (PMC10101851; doi:10.1038/s41388-023-02611-y)
Supplement: Supplementary file 2 — Supplementary figures [file 41388_2023_2611_MOESM2_ESM.pdf]

Fig. S1

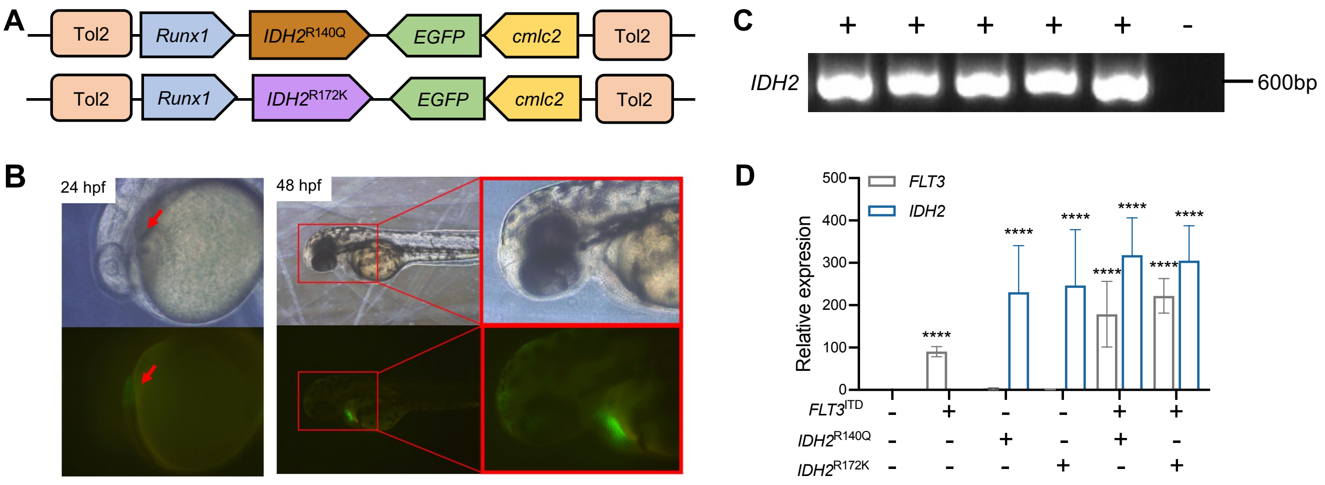
A
Tol2
Runx1
IDH2R140Q
EGFP
cmlc2
Tol2
Tol2
Runx1
IDH2R172K
EGFP
cmlc2
Tol2
B
24 hpf
48 hpf
C
+ + + + + -
IDH2
600bp
D
Relative expresion
500
400
300
200
100
0
FLT3
IDH2
****
FLT3ITD - + - - + +
IDH2R140Q - - + - + -
IDH2R172K - - - + - +

Fig. S2

A

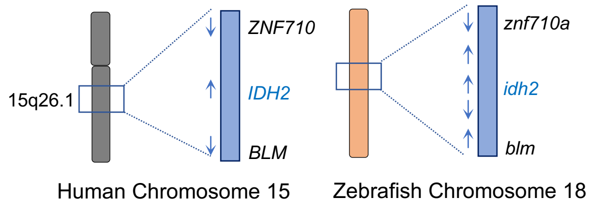

B

Location of *IDH2*[R140Q] — Location of *IDH2*[R172K]

```
Zebrafish Idh2  MAGYLKVLSSLSRSAATLSKSPAVLAPACQSLQQRNYADKRIQVAQPVVEMDGDEMTRIIWEFIKEKLILTNVNVDLKYYDLGLPYRDQTDDQVTIDSAIATKKYNVAVKCATITPDEAR  120
Human IDH2      --------------------------------------------------MDGDEMTRIIWQFIKEKLILPHVDIQLKYFDLGLPNRDQTDDQVTIDSALATQKYSVAVKCATITPDEAR  120
                                                                  ***********:******** :*:::***:***** *************:**:**.**************

Zebrafish Idh2  VEEFKLKKMWKSPNGTIRNILGGTVFREPIICKNIPRLVPGWTQAITIGRHAFGDQYKATDFVVNQPGKFKMVFTPADGSKNKEWEVYDFPGGGCGMGMYNTDESITGFAHSCFQYAIQK  240
Human IDH2      VEEFKLKKMWKSPNGTIRNILGGTVFREPIICKNIPRLVPGWTKPITIGRHAHGDQYKATDFVADRAGTFKMVFTPKDGSGVKEWEVYNFPAGGVGMGMYNTDESISGFAHSCFQYAIQK  190
                ******************************************: *******.**********.:: *.******* ***  ******:**.** ***********:*************

Zebrafish Idh2  KWPLYMSTKNTILKAYDGRFKDIFQDIFEKNYKPEFDKLKIWYEHRLIDDMVAQVLKSSGAFVWACKNYDGDVQSDILAQGFGSLGLMTSVLVCPDGKTIEAEAAHGTVTRHYREHQKGR  360
Human IDH2      KWPLYMSTKNTILKAYDGRFKDIFQEIFDKHYKTDFDKNKIWYEHRLIDDMVAQVLKSSGGFVWACKNYDGDVQSDILAQGFGSLGLMTSVLVCPDGKTIEAEAAHGTVTRHYREHQKGR  310
                *************************:**:*:** :*** ********************.***********************************************************

Zebrafish Idh2  PTSTNPIASIFAWTRGLEHRGKLDGNPDLIKFSQTLERVCVETVESGVMTKDLAGCIHGLANCKLNEHYVNTTDFLDAIKTNLDKALGK-  449
Human IDH2      PTSTNPIASIFAWTRGLEHRGKLDGNQDLIRFAQMLEKVCVETVESGAMTKDLAGCIHGLSNVKLNEHFLNTTDFLDTIKSNLDRALGRQ  400
                ************************** ***:*:* **:*********.************:* *****::*******:**:***:***:
```

Fig. S3

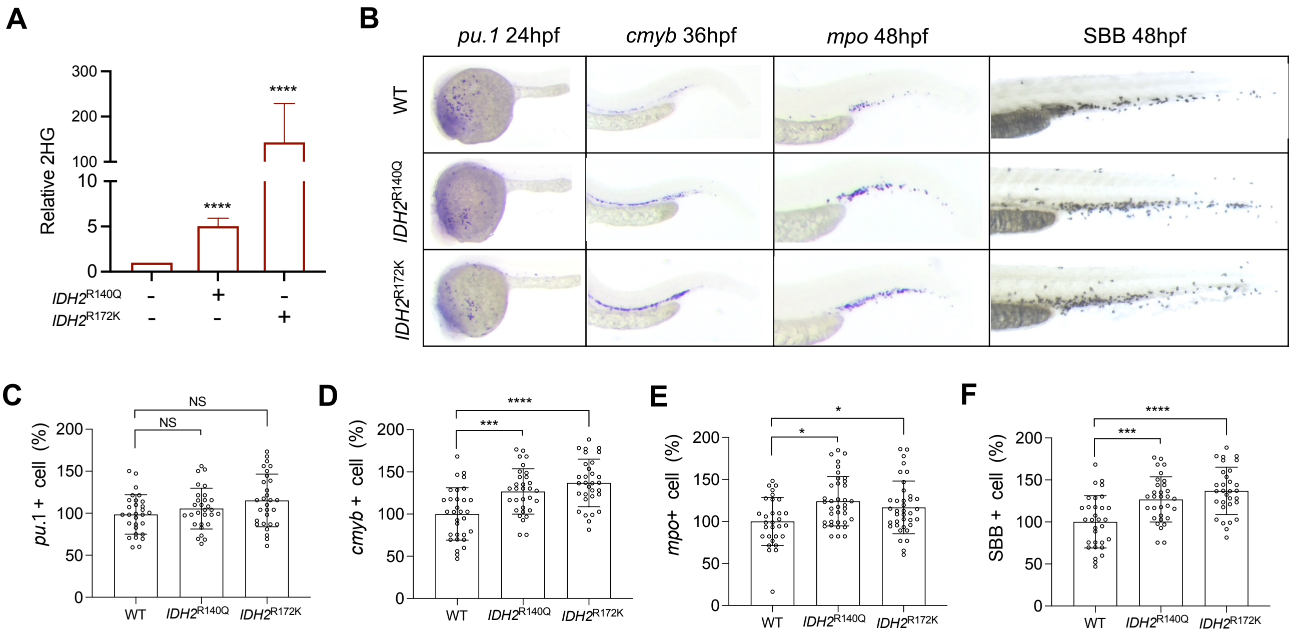
A
B
Relative 2HG
300
200
100
10
5
0
****
****
IDH2R140Q - + -
IDH2R172K - - +
pu.1 24hpf
cmyb 36hpf
mpo 48hpf
SBB 48hpf
WT
IDH2R140Q
IDH2R172K
C
D
E
F
pu.1 + cell (%)
cmyb + cell (%)
mpo+ cell (%)
SBB + cell (%)
NS
***
****
*
200
150
100
50
0
WT
IDH2R140Q
IDH2R172K

Fig. S4

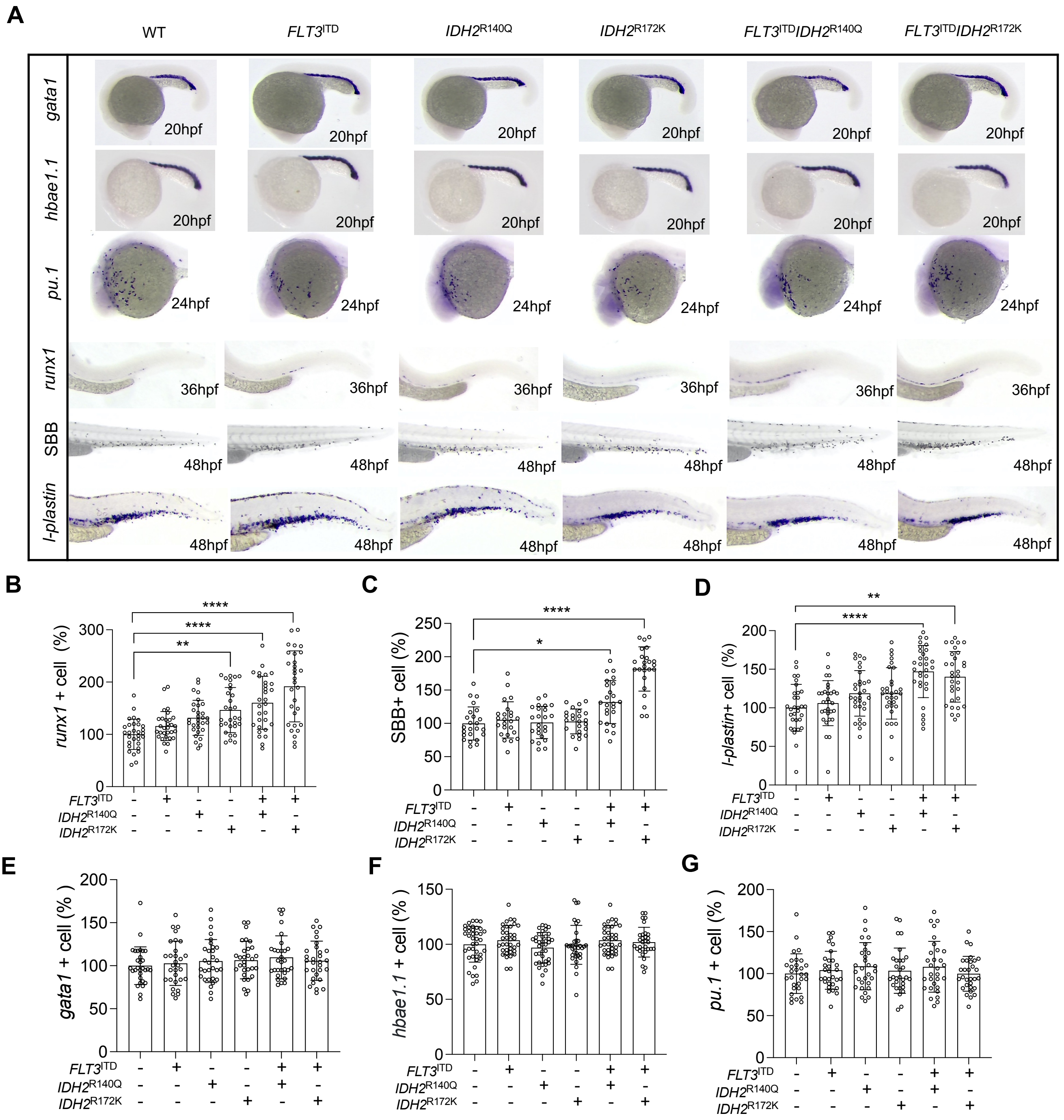
A
WT
FLT3ITD
IDH2R140Q
IDH2R172K
FLT3ITDIDH2R140Q
FLT3ITDIDH2R172K
gata1
hbae1.1
pu.1
runx1
SBB
l-plastin
20hpf
24hpf
36hpf
48hpf
B
runx1 + cell (%)
C
SBB+ cell (%)
D
l-plastin+ cell (%)
E
gata1 + cell (%)
F
hbae1.1 + cell (%)
G
pu.1 + cell (%)
FLT3ITD
IDH2R140Q
IDH2R172K

Fig. S5

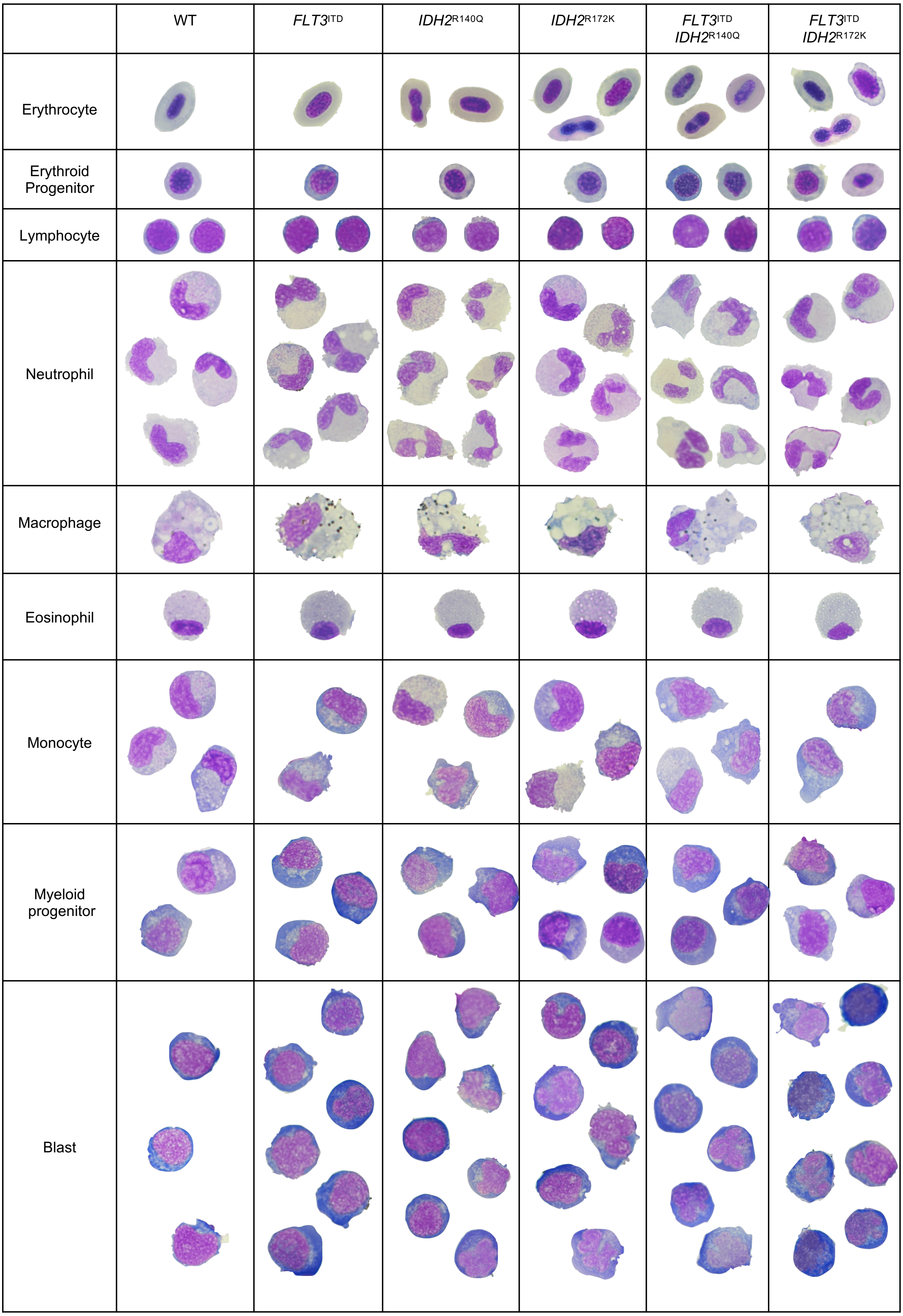
WT
FLT3ITD
IDH2R140Q
IDH2R172K
FLT3ITD IDH2R140Q
FLT3ITD IDH2R172K
Erythrocyte
Erythroid Progenitor
Lymphocyte
Neutrophil
Macrophage
Eosinophil
Monocyte
Myeloid progenitor
Blast

Fig. S6

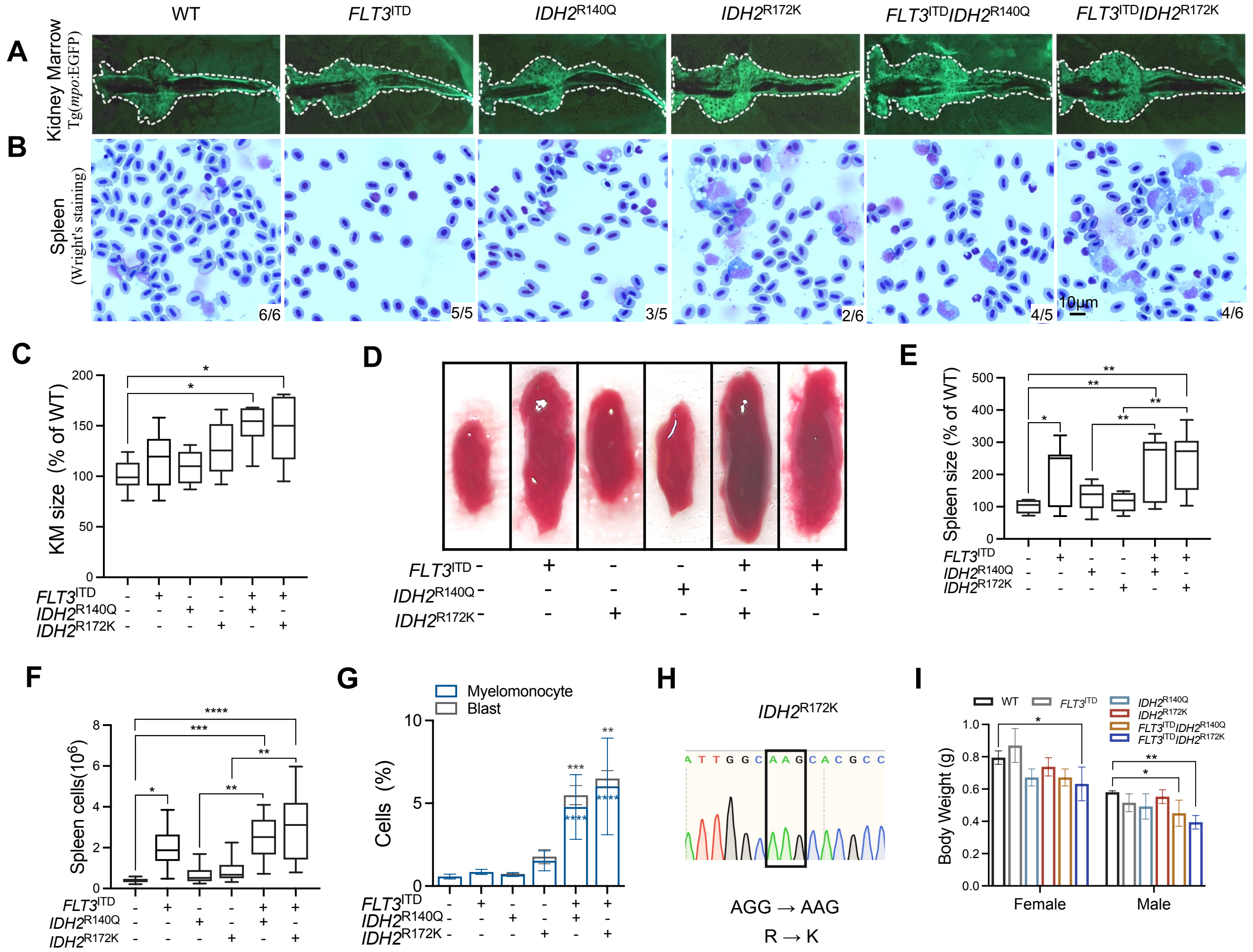

A
B
WT
FLT3ITD
IDH2R140Q
IDH2R172K
FLT3ITDIDH2R140Q
FLT3ITDIDH2R172K
Kidney Marrow Tg(mpo:EGFP)
Spleen (Wright's staining)
6/6
5/5
3/5
2/6
4/5
10μm
4/6
C
KM size (% of WT)
D
E
Spleen size (% of WT)
F
Spleen cells(10^6)
G
Myelomonocyte
Blast
Cells (%)
H
IDH2R172K
AGG → AAG
R → K
I
Body Weight (g)
Female
Male

Fig. S7

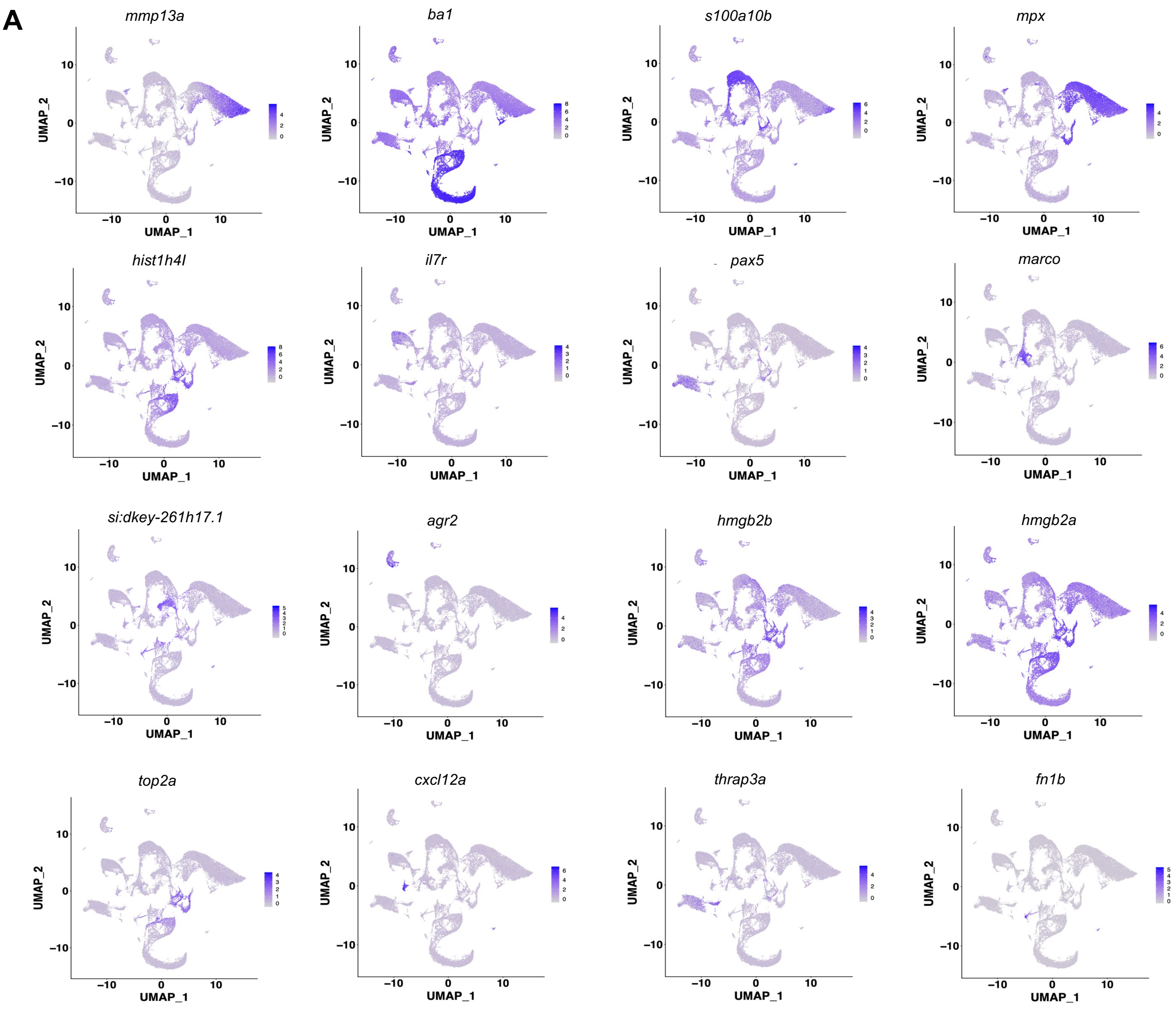
A
mmp13a
ba1
s100a10b
mpx
hist1h4l
il7r
pax5
marco
si:dkey-261h17.1
agr2
hmgb2b
hmgb2a
top2a
cxcl12a
thrap3a
fn1b
UMAP_1
UMAP_2

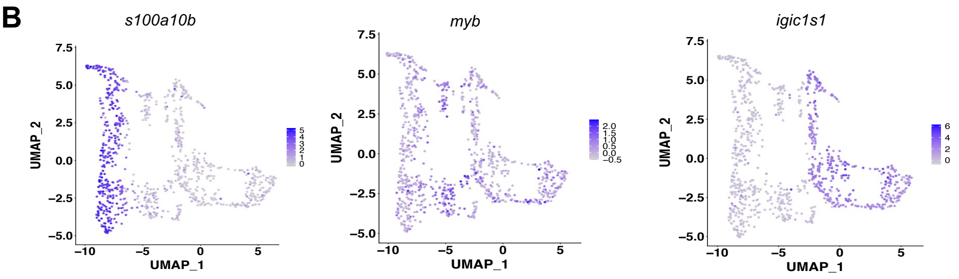
B
s100a10b
myb
igic1s1
UMAP_1
UMAP_2

Fig.S8

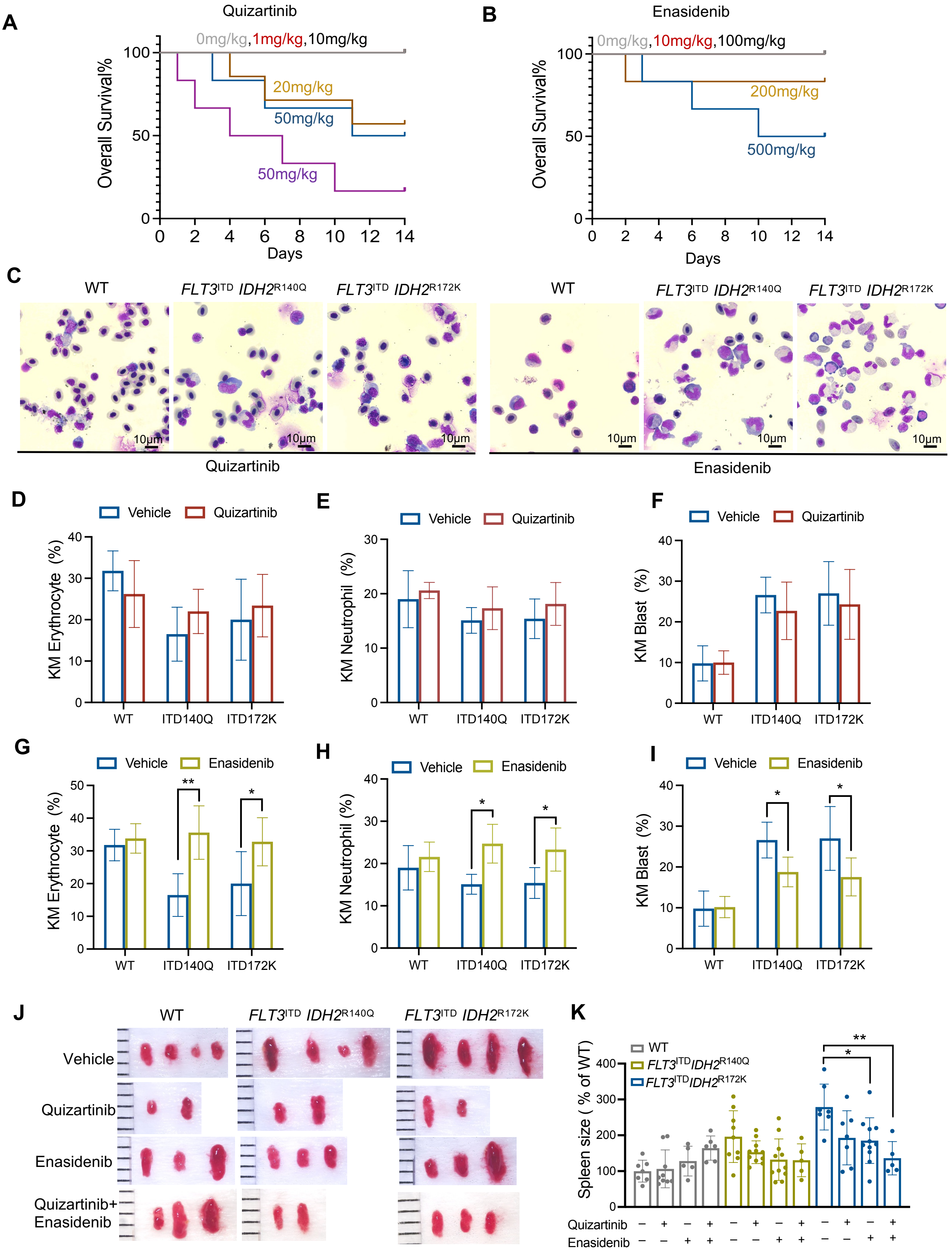
A
Quizartinib
0mg/kg,1mg/kg,10mg/kg
20mg/kg
50mg/kg
50mg/kg
Overall Survival%
Days
B
Enasidenib
0mg/kg,10mg/kg,100mg/kg
200mg/kg
500mg/kg
Overall Survival%
Days
C
WT
FLT3ITD IDH2R140Q
FLT3ITD IDH2R172K
10μm
Quizartinib
Enasidenib
D
E
F
Vehicle
Quizartinib
KM Erythrocyte (%)
KM Neutrophil (%)
KM Blast (%)
WT
ITD140Q
ITD172K
G
H
I
Vehicle
Enasidenib
J
WT
FLT3ITD IDH2R140Q
FLT3ITD IDH2R172K
Vehicle
Quizartinib
Enasidenib
Quizartinib+
Enasidenib
K
WT
FLT3ITDIDH2R140Q
FLT3ITDIDH2R172K
Spleen size (% of WT)
Quizartinib
Enasidenib
